# The age related markers lipofuscin and apoptosis show different genetic architecture by QTL mapping in short-lived Nothobranchius fish

**DOI:** 10.18632/aging.100660

**Published:** 2014-05-12

**Authors:** Enoch Ng'oma, Kathrin Reichwald, Alexander Dorn, Michael Wittig, Tobias Balschun, Andre Franke, Matthias Platzer, Allesandro Cellerino

**Affiliations:** ^1^ Biology of Ageing, Leibniz Institute for Age Research – Fritz Lipmann Institute, 07745 Jena, Germany; ^2^ Genome Analysis, Leibniz Institute for Age Research – Fritz Lipmann Institute, 07745 Jena, Germany; ^3^ Institute of Clinical Molecular Biology, Christian-Albrechts-University, 24105 Kiel, Germany; ^4^ Hufeland Klinikum Mühlhausen, Institut für Infektiologie und Pathobiologie, 99974 Mühlhausen, Germany; ^5^ Neurobiology Laboratory, Scuola Normale Superiore, 56124 Pisa, Italy

**Keywords:** Nothobranchius, lifespan, lipofuscin, apoptosis, quantitative trait loci, aging

## Abstract

Annual fish of the genus Nothobranchius show large variations in lifespan and expression of age-related phenotypes between closely related populations. We studied *N. kadleci* and its sister species *N. furzeri* GRZ strain, and found that *N.kadleci* is longer-lived than the *N. furzeri*. Lipofuscin and apoptosis measured in the liver increased with age in *N. kadleci* with different profiles: lipofuscin increased linearly, while apoptosis declined in the oldest animals. More lipofuscin (*P* < 0.001) and apoptosis (*P* < 0.001) was observed in *N. furzeri* than in *N. kadleci* at 16w age. Lipofuscin and apoptotic cells were then quantified in hybrids from the mating of *N. furzeri* to *N. kadleci*. F_1_ individuals showed heterosis for lipofuscin but additive effects for apoptosis. These two age-related phenotypes were not correlated in F_2_ hybrids. Quantitative trait loci analysis of 287 F_2_ fish using 237 markers identified two QTL accounting for 10% of lipofuscin variance (*P* < 0.001) with overdominance effect. Apoptotic cells revealed three significant- and two suggestive QTL explaining 19% of variance (*P* < 0.001), showing additive and dominance effects, and two interacting loci. Our results show that lipofuscin and apoptosis are markers of different age-dependent biological processes controlled by different genetic mechanisms.

## INTRODUCTION

Aging is defined as age-dependent increase in mortality risk. In complex organisms, this is the result of progressive functional impairment of organ systems and is accompanied by a myriad of aging-associated phenotypes that can be expressed in different combinations in individuals of same chronological age (e.g. [1]). In many cases, it remains unclear to what extent these phenotypes correlate with functional impairment and therefore with individual mortality risk or rather with chronological age. Lipofuscin, or the “age pigment”, is the prototypical and most studied age-dependent marker. Lipofuscin granules accumulate in post-mitotic cells of many organisms and its rate of accumulation was linked to age-dependent mortality in *C. elegans* [2] and arthropods [3]. Lipofuscin load in mice showed the best correlation with both chronological age and age-dependent gene expression out of 18 pathological parameters [1]. Lipofuscin is a highly cross-linked, degradation-resistant and non-exocytosable aggregate comprising oxidized proteins, lipids and metal elements which accumulate mainly in the lysosomes of post-mitotic cells and is detected by autofluorescence [4]. Lipofuscin accumulation reduces lysosomal autophagic capacity by acting as enzyme sink consequently depressing recycling capacity and increasing build-up of damaged macromolecules [5] and may be related to loss of protein homeostasis [6] that is a hallmark of aging [7].

Apoptosis is a critical process for controlling cell numbers during development and tissue homeostasis and is the key pathological mechanism in many degenerative diseases. Apoptosis may be linked to lysosomes and lipofuscin in that oxidant-induced lysosomal rupture can cause leakage of lysosomal cathepsin D and mitochondrial cytochrome *c*, consequently activating caspases, which trigger apoptosis [8, 9]. In addition, cytokeratin-18 (CK-18), a marker for apoptosis, was reported in human cardiac lysosomes containing large amounts of lipofuscin [10].

The African annual killifish genus Nothobranchius has emerged as a tractable model system for the study of vertebrate aging because of short lifespan and rapid expression of age-related phenotypes [11-14]. Captive populations of *N. furzeri* show reproducible variations in lifespan and expression of age-related histological traits, including lipofuscin accumulation and apoptosis [15]. Comparison of species pairs demonstrated that duration of the water habitat correlates positively with lifespan and negatively with accumulation of lipofuscin in captive population [16]. Pairs of species and strains with different aging rates provide a tractable model to study the genetic architecture underlying the evolution of senescence by means of quantitative trait loci (QTL) analysis. Simple traits [17] and QTL for lifespan [18] were previously mapped in an intraspecific cross of short- and longer-lived *N. furzeri* strains. Here, we took a complementary approach by mapping QTL affecting variation in the expression of age-related traits in individuals of the same chronological age originating from a cross between *N. furzeri* GRZ strain and its sister species, *N. kadleci*. *N. furzeri* GRZ is a very short-lived teleost and shows typical aging-related phenotypes including physiological and cognitive decline, and expression of aging-related biomarkers [12, 14]. We characterized and discuss: 1) the lifespan and genetic variability of *N. kadleci* in relation to the short-lived *N. furzeri* strain GRZ, 2) the differential expression of lipofuscin and apoptosis in *N. kadleci* and *N. furzeri* GRZ, and 3) the heritability patterns and QTL for these two phenotypes in F_2_ fish.

## RESULTS

### Lifespan of *N. kadleci*

**Figure 1:**
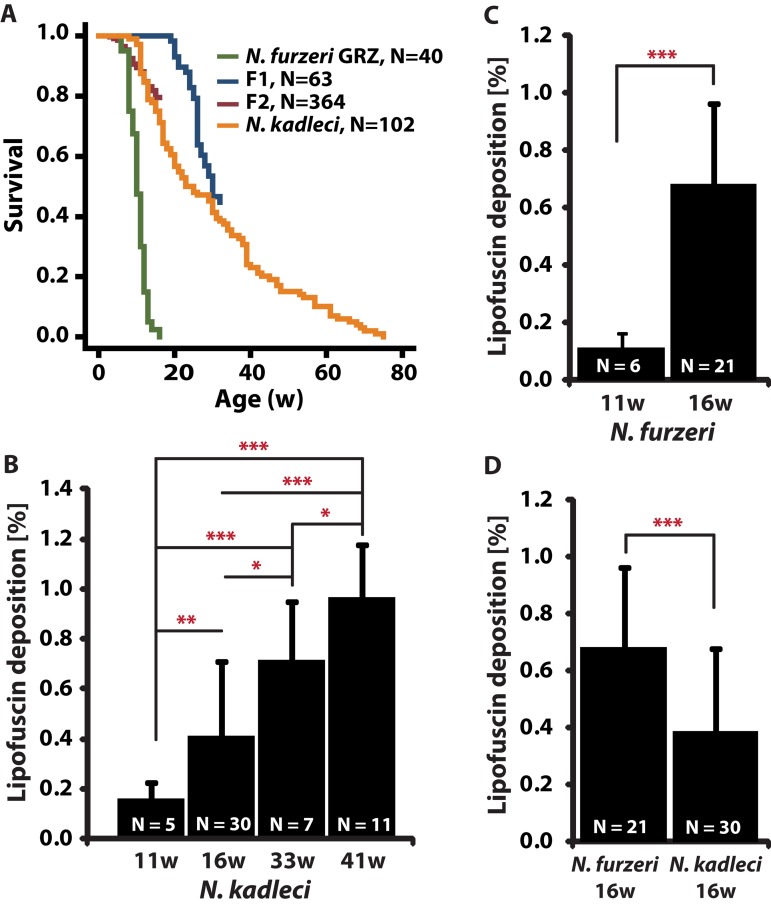
Lifespan and lipofuscin deposition (LFD) with age in *N. furzeri* GRZ and *N. kadleci* (**A**) Kaplan Meier estimates of survival for populations of *N. furzeri* GRZ, F_1_, F_2_ and *N. kadleci*. (**B**-**D**) Quantification of LFD: (B) *N. kadleci* at four time points. (**C**) *N. furzeri* GRZ at two time points. (D) *N. furzeri* GRZ and *N. kadleci* at the same chronological age. Error bars represent standard deviation; significant differences are indicated by asterisks: (*) = *P* < 0.05, (**) = *P* < 0.01 and (***) = *P* < 0.001, *T*-test.

To describe the captive lifespan of *N. kadleci*, three separate experiments were followed between 2009 and 2013. In each experiment, hatchlings from the same batch of eggs were included in the study. *N. kadleci* captive lifespan was significantly longer in comparison to *N. furzeri* GRZ (*P* < 0.001, Log-Rank test) with mean- and 10%-survival of 27 weeks vs. 12 weeks and 60 weeks vs. 17 weeks, respectively (Fig. 1a). *N. kadleci* showed high levels of initial mortality resulting in a very progressive decay of survivorship that is replicated in the different cohorts. There was no difference in survival between genders.

### Characterization of lipofuscin deposition in *N. kadleci*

Lipofuscin deposition (LFD) in *N. kadleci* was quantified at 4 time points (11-, 16-, 33- and 41-weeks) and increased almost linearly throughout life (R^2^ = 0.39, Fig. 1b). LFD in *N. furzeri* GRZ increased 6.2-fold from 11 weeks to 16 weeks (Mann-Whitney test, *P* < 0.001) (Fig. 1c). At 16 weeks, GRZ fish had nearly twice as much (1.7-fold) LFD as *N. kadleci* (Fig. 1d). Since LFD increased linearly with age, we estimated the “equivalent age” for a 16w old *N. furzeri*-GRZ to be approximately 26 weeks in *N. kadleci*.

### Characterization of apoptotic cell fraction in *N. kadleci*

**Figure 2:**
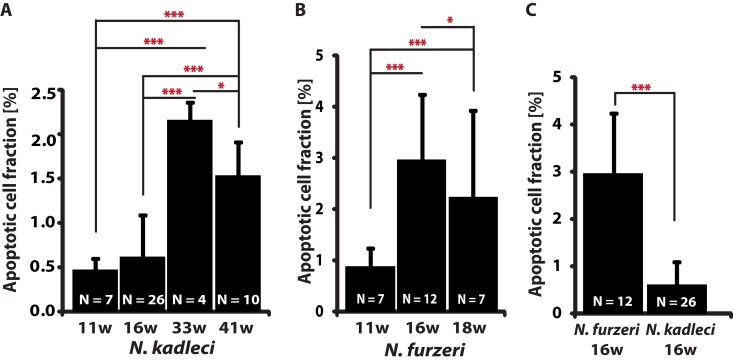
Apoptotic cells fraction (ACF) change with age in *N. furzeri* GRZ and *N. kadleci* (**A**) *N. kadleci* at four time points. (**B**) *N. furzeri* GRZ at three time points. (**C**). Comparison in age-matched samples of both species. Error bars represent standard deviation; significant differences are indicated by asterisks: (*) = *P* < 0.05, (**) = *P* < 0.01 and (***) = *P* < 0.001, *T*-test.

We determined the apoptotic cell fraction (ACF) from fluorescent images acquired from TUNEL-stained paraffin-embedded liver tissue sections of 11-, 16-, 33- and 41-weeks old *N. kadleci* and 11-, 16-, and 18-weeks old *N. furzeri* GRZ. ACF did not show a monotonous age-dependent increase: it increased 3.5-fold between 16 and 33 weeks (*P* < 0.001) but dropped 33% between 33- and 41 weeks (*P* < 0.05, Fig. 2a). Similarly, GRZ showed a 4-fold increase in ACF between 11- and 16 weeks (*P* < 0.01), and between 11- and 18 weeks, a 23% decline (Fig. 2b). At age 16 weeks there was a 4.8-fold difference in ACF between *N. kadleci* and GRZ (*P* < 0.001) (Fig.2c). Interestingly, although ACF increased in an age-dependent manner in both species, 1) both showed that individuals attaining very old age had lower ACF, and 2) *N. furzeri* GRZ showed steeper age-dependent increase as compared to *N. kadleci*. ACF was further significantly correlated between 16w old *N. kadleci* and F_1_ offspring (r = 0.68; *P* < 0.05).

### The mapping population

**Figure 3:**
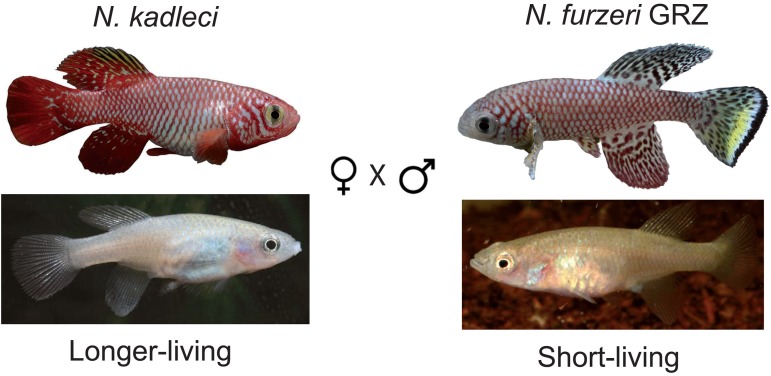
The mating scheme A female of the longer-living species *N. kadleci* was mated to a male of the short-lived species *N. furzeri* GRZ strain, resulting into 35 F_1_ individuals. Sibling mating in 13 families of the F_1_ gave rise to 364 F_2_ fish. Of these, 293 (81%) were successfully scored for phenotypes and genotyped at 16 weeks of age.

We generated a mapping population from an inter-cross between one male of the shortest living *N. furzeri* strain GRZ and one female of the longer-living species *N. kadleci* (Fig. 3). Of the resulting 34 F_1_ fish, 13 sister-brother breeder pairs produced a total of 364 F_2_ progeny surviving at least 5 weeks. All F_2_ fish that reached age 16 weeks were sacrificed for histological analysis (287 fish, 81% survivorship).

### Lifespan of hybrid fish

Unlike *N. kadleci*, with high initial mortality and progressive decay in survivorship, a result that was replicated in three experiments, the F_1_ hybrid fish, had a median lifespan that was significantly longer (30 weeks vs. 27 weeks; Log-Rank, *P* < 0.05). A comparison with the F_2_ at 50% survival was not possible, as all surviving F_2_ were sacrificed at the age of 16 weeks for histological analysis.

### Linkage analysis

To obtain markers for QTL, we sequenced in *N. kadleci* 441 loci polymorphic in *N. furzeri* (Reichwald *et al.* 2009) and identified 287 single nucleotide variations (SNVs) polymorphic in parents of the present cross. Additional 54 SNVs were identified from alignment of *N. kadleci* genomic sequences of protein-coding loci to those of *N. furzeri*. These 341 markers were used to genotype 342 F_2_ individuals resulting into 258 SNVs (76%) being successfully genotyped. The genotype data matrix contained 253 markers, 341 individuals and was 96.2% complete (i.e. 3.8% missing genotypes). A genetic linkage map of 1705.1 cM across 20 linkage groups (LGs) was thereafter obtained, containing 237 markers, whose order was largely consistent with a previous map obtained in intraspecific crosses of *N. furzeri* [18]. Several diagnostic analyses of genotypes were made to assess data quality and map properties. Genotyping error rate was one important diagnostic analysis undertaken, and this revealed a log_10_ likelihood error estimate of 0.3% (data not shown).

### Segregation ratio distortion at SNV markers

Forty-two (16.6%) of linked markers significantly deviated from the expected Mendelian frequencies of 1:2:1 (χ^2^, *P* < 0.05). Of these, 28 showed higher frequency of the paternal (*N. furzeri* GRZ) alleles, 12 of the maternal (*N. kadleci*) alleles, and 2 of the heterozygous genotypes (Tables S3). Overall, there was preferential transmission of paternal alleles from *N. furzeri* GRZ (χ^2^=31.0, *P* < 0.0001). Distortion of some sort was suggested in 14 (70%) of the linkage groups (LGs). Evidence for segregation distortion was mapped as distortion QTL on –log10(*P*) scale alongside marker genotype frequencies (Fig. S1). We interpret this datum as a sign of incipient genetic incompatibility between these two closely-related, but separate species.

### Heterosis in LFD in hybrid populations

**Figure 4:**
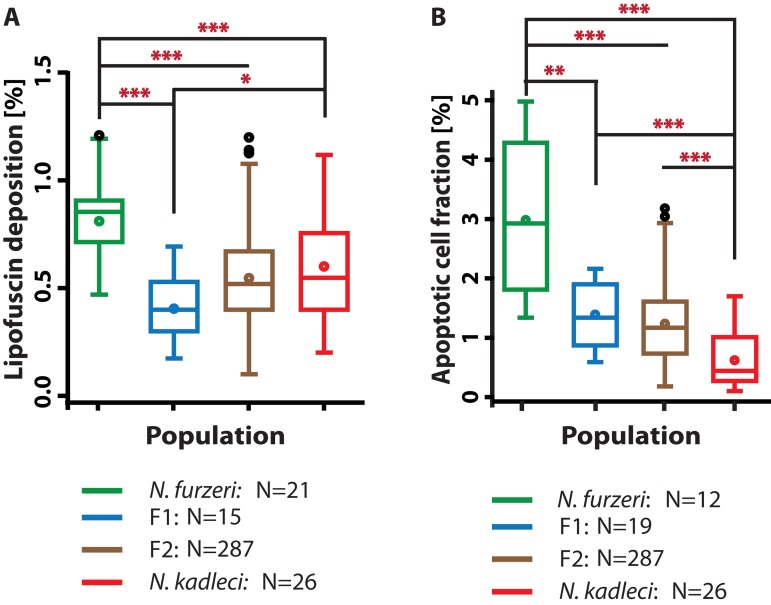
LFD and ACF in hybrids compared with *N. furzeri* GRZ and *N. kadleci* (**A**) LFD enrichment at 16w in F_1_ and F_2_ hybrids compared to parental populations at 16 weeks (**B**) ACF at 16w in F_1_ and F_2_ hybrids and parental populations at 16 weeks. Boxes represent 25^th^ – 75^th^ quartiles, whiskers show maximum and minimum data points excluding outliers (circles above whiskers), line in box is median, circle in box is mean. Significant differences are indicated by asterisks: (*) = *P* < 0.05, (**) = *P* < 0.01 and (***) = *P* < 0.001, Mann-Whitney U-test. N is number of individuals evaluated.

We compared LFD in the F_1_ and F_2_ generations at 16 weeks with parental populations and found that F_1_ hybrids exhibited significantly reduced LFD compared with both *N. furzeri* GRZ (*P* < 0.001) and *N. kadleci* (*P* < 0.05) by Mann-Whitney test (Fig. 4a). F_2_ fish had significantly fewer LFD compared to GRZ (*P* < 0.001); they did not differ from *N. kadleci* and the F_1_.

### ACF in hybrid populations showed additive segregation

We compared ACF in 16 weeks old parental and offspring populations (Fig. 4b). ACF in the F_1_ and F_2_ were significantly lower than in GRZ (*P*<0.01 and *P*<0.001, respectively, Mann-Whitney test), but higher than *N. kadleci* (*P*<0.001). Hybrid distributions did not differ significantly from each other. The distribution of values in F_2_ (N = 287) showed the expected greater variance.

### Two QTL for LFD in *Nothobranchius* spp

**Figure 5:**
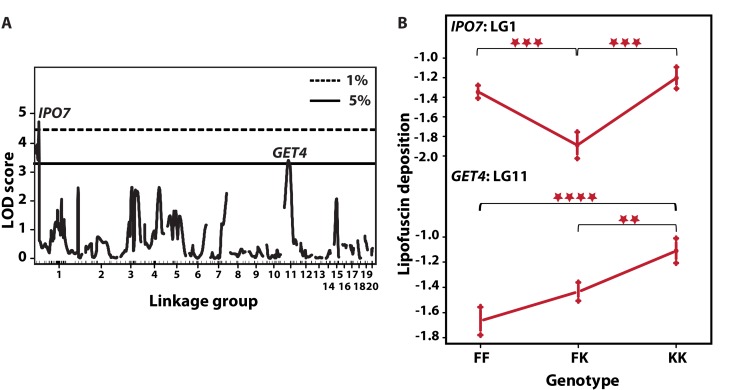
QTL for LFD and their allelic effects (**A**) Single-QTL genome-wide scan. LOD scores (*y*-axis) are plotted against genetic distance (cM, *x*-axis). Horizontal lines depict data-derived significant LOD score thresholds (permutation test): dotted line, *P < 0.01* and solid line *P < 0.05.* Two significant peaks appear on LG1 at marker *IPO7* with LOD 4.40 and LG11 at *GET4*, LOD 3.44. Linkage groups are shown on the *x*-axis and markers depicted with inner tick marks. (**B**) Plot of estimated phenotype averages at markers *IPO7* and *GET4*. The *y*-axis indicates phenotype values on logarithmic scale. The *x*-axis depicts the three genotypes at each marker – FF refers to F_2_ homozygous for *N. furzeri* GRZ allele, FK stands for heterozygous F_2_ and KK for F_2_ homozygous for the *N. kadleci* allele. Bars at each genotype are standard error of mean. Significant differences in phenotype mean between genotypes are shown by asterisks (**) =*P*<0.01, (***) = *P*<0.001, (****) = *P*<0.0001.

Prior to QTL analysis, we tested whether LFD was affected by gender, family, and physical attributes including fish mass and total length. LFD was not correlated with any of these variables either singularly, or with adjustment. Then, we scanned for QTL influencing LFD by fitting the standard interval mapping model assuming a single LFD QTL per LG. This model compares the likelihood that one QTL affecting the phenotype is present on a given LG with the likelihood that there is no QTL on that LG, in genomic intervals of 1 cM (in R/qtl *scanone* function). The model revealed two QTL with a cumulative LOD score of 8.1 and accounting for 10.1% in LFD variation (Table 1). The two QTL with genome-wide significance were located on LG1 (LOD score of 4.87, *P* < 0.01, permutation test) and LG11 (LOD score of 3.31, *P* < 0.05) (Fig. 5a). On LG1, the terminal marker *IPO7* (Importin-7) formed QTL peak with Bayes LOD interval extending to *DHX38*. On LG11, the QTL mapped on marker *GET4*, and Bayes interval extended between *ANXA11* to *PSMC5*. A multi-QTL scan retained peaks on LG1 and LG11, while revealing a third peak marginally reaching significance on marker *UBE2D2* on LG4. However, this peak did not substantially improve explained variance in LFD. There was no evidence of interaction between the loci. Analysis of allelic effects showed overdominance in F_2_ fish heterozygous at *IPO7* on LG1 (Fig. 5b). Contrary to expectation, both QTL showed “transgressive” effects in that the *N. kadleci* homozygous genotype (KK) resulted in greater LFD than the *N. furzeri* homozygous genotype (FF).

**Table 1:** Estimated QTL effects for lipofuscin deposition. The effects of an additive model without assuming interaction are shown, including additive and dominance effects for each QTL

LG	QTL(cM)ǂ	LOD	%Var.†	*F*	*P*-val.^ˆ^	BayesInt‡	a (se)^ˇ^	d (se)^ˇ^	Marker^˚^
1	2.0	4.87	5.29	8.20	***	0.0-16.4	0.082 (0.064)	-0.601 (0.150)	*IPO7*
11	39.0	3.31	4.30	6.67	**	0.00-50.1	0.260 (0.073)	-0.047 (0.103)	*GET4*

LG, linkage group

ǂ position of QTL in centimorgan

† variance explained by QTL in F2 phenotype

‡ Bayes confidence intervals (Manichaikul *et al.* 2006)

˚ Marker nearest to the cM position at which the greatest *F*-value was observed

ˆ *P* values of the F-statistic: ‘***’< 0.001, ‘**’< 0.01, ‘*’< 0.05

ˇ Additive (a) and dominance (d) effects with standard error in parentheses calculated from *ln*-transformed values, and direction shown as *N. kadleci* allele effect.

### Five QTL affecting ACF

**Figure 6:**
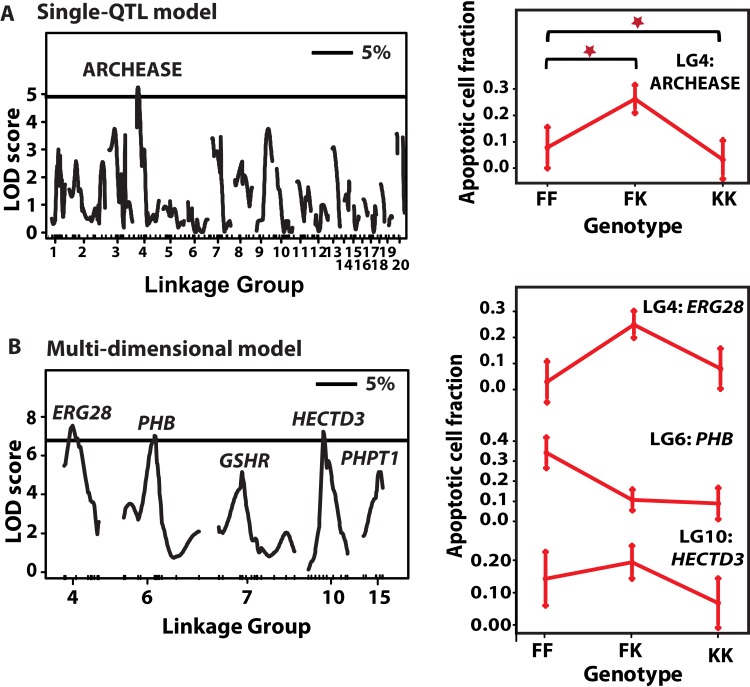
QTL for ACF and their allelic effects (**A**) *Left*: Single-QTL genome-wide scan. LOD scores (*y*-axis) are plotted against genetic distance (cM, *x*-axis) Horizontal line depict data-derived significant LOD score threshold (*P < 0.*05, permutation test). Linkage groups are shown on the *x*-axis and markers depicted with inner tick marks. One significant LOD peak is appeared on LG4 at marker ARCHEASE with LOD 4.78. (**A**) *Right*: QTL effects at LG4 QTL. The *x*-axis depicts the three genotypes at each marker – FF refers to F_2_ homozygous for *N. furzeri* GRZ allele, FK stands for heterozygous F_2_ and KK for F_2_ homozygous for the *N. kadleci* allele. Bars at each genotype are standard error of mean. (**B**) *Left*: Multi-QTL scan. Significant LOD score threshold, *P* < 0.05. Three significant LOD peaks were detected on LG4 (*ERG28*), LG6 (*PHB*), and LG10 (*HECTD3*), and two suggestive QTL on LG7 (*GSHR*) and LG15 (*PHPT1*). (**B**) *Right*: Effect plots of the three significant QTL.

As for LFD, ACF were not correlated with gender,mass, total length and lineage. A single QTL fit identified one peak with genome-wide significance at LG4 marker ARCHEASE, explaining 9% of the variation in ACF (Fig. 6a). We then carried out step-wise addition of cofactors to the initial locus to build multi-QTL models affecting ACF. Models with three to seven factors accounted for between 16% and 22% of variation in ACF. A three-QTL model contained QTL on LGs 4, 6 and 10, all of which were significant *P* < 0.05. A five-factor model included two additional suggestive QTL on LGs 7 and 15 (*P* < 0.1, Fig. 6b). The QTL with genome-wide support on LG4 appeared in almodels explored, but the LOD peak shifted to marker *ERG28* in all the models. Peak markers in other QTL included *PHB* on LG6 and *HECTD3* on LG10. Suggestive QTL peaked on *GSHR* and *PHPT1* at LG 7 and LG 15, respectively. The QTL on LG7 seemed to comprise two separate loci, one peaking at *PLXNA4* and another on *GSHR,* with opposite allelic effects.

QTL effects for individual loci are reported in Table 2, Figure 6 (right panel) and Fig. S2. Overall, two results were noted. First, the KK genotype generally showed lower levels of ACF than the FF genotype at most loci. Second, among the heterozygote genotypes FK, two sets of allelic effects were seen, a) *ERG28*, *HECTD3*, and *PLXNA4* had greater ACF compared to both homozygote genotypes, and b) at *PHB*, *PHPT1* and *GSHR*, the heterozygotes had ACF levels similar to the KK genotype. (See effect plots for *PLXNA4*, *PHPT1* and *GSHR* in Fig. S2.) In addition, significant dominance-additive interaction (d:a = 1.07) was detected between QTL on LG10 and LG4 (*P* < 0.001).

**Table 2:** Estimated QTL effects for apoptotic cell fraction (ACF). Results for the multiple QTL additive model are shown, including additive and dominance effects for each QTL

LG	QTL, cMǂ	LOD	%Var.†	*F*	*P*-val.^ˆ^	BayesInt‡	Additive (se)^ˇ^	Dominance (se)^ˇ^	Peak marker^˚^
**Additive**									
4	43	8.4	11.9	3.8	***	0.0-57.3	0.00(0.06)	0.22(0.08)	*ERG28*
10	33	5.7	7.1	4.18	***	24.1-40.6	0.16 (0.11)	0.35(0.15)	*HECTD3*
15	24	5.4	5.7	3.7	**	19.2-28.3	0.06(0.09)	0.05 (0.13)	*PHPT1*
6	45	2.1	2.9	4.5	*	21.1-55.7	−0.15(0.05)	−0.09(0.08)	*PHB*
7	2	0.9	1.2	0.65	*	0.0-105.2^ˇ^	0.02 (0.06)	0.16 (0.08)	*GSHR*
**Interaction**						a:a	d:d	a:d	d:a
10:4	56:43	6.7	7.0	5.5	***	0.01(0.13)	−0.01(0.33)	0.35(0.16)	1.07(0.23)

LG, linkage group

ǂ position of QTL in centimorgan

† variance explained by QTL in F2 phenotype

‡ Bayes confidence intervals (Manichaikul *et al.* 2006)

˚ Marker nearest to the cM position at which the greatest *F*-value was observed

ˆ *P* values of the F-statistic: ‘***’< 0.001, ‘**’< 0.01, ‘*’< 0.05

ˇ Additive (a) and dominance (d) effects with standard error in parentheses calculated from *ln*-transformed values, and direction shown as *N. kadleci* allele effect. Effect interactions are shown with (:)

### Relationship between LFD and ACF

LFD and ACF were both age-dependent in the parental generations. Interestingly, these traits did not correlate with each other in F_2_ fish (r = −0.041). Greater intra-familial dispersion was observed in LFD compared to ACF (Fig. S3). Indeed, we found no significant QTL shared by these two phenotypes. However, suggestive QTL for LFD at marker *UBE2D2* (P53-regulated ubiquitin-conjugating enzyme) was located at one end of LG4 (at 93.4 cM) and both peak markers for apoptosis (ARCHEASE and *ERG28*), were located on the opposite end of the LG (1.5 cM and 43.1 cM; respectively).

## DISCUSSION

### Survivorship of *N. kadleci*

Previous studies described lifespan of captive populations

of several Nothobranchius species and strains [11-13, 15, 16, 19]. One important finding of these studies is that different Nothobranchius species and strains of *N. furzeri* show differences in lifespan that remain stable over many captive generations. Our data showed that *N. kadleci* is a longer-living species as compared to the *N. furzeri* GRZ strain in terms of both mean (27w vs. 12w) and maximum (60w vs. 17w) lifespan. The survivorship curve of *N. kadleci* (Fig. 2a) showed high levels of initial mortality with rather progressive reduction of survival - a combination of Type I (negatively skewed rectangular) and Type II (diagonal) survivorship [20], that is different from typical survivorship of *N. furzeri* or other Nothobranchius species [16, 19] and more typical for *r*-selected species i.e. organisms living in unstable habitats that grow rapidly, reproduce early, and typically produce large numbers of offspring with large early life mortality [21]. At the present, it is unclear whether this reflects genetic heterogeneity of the wild population or stochastic processes.

### LFD in *N. kadleci* and hybrid populations

LFD increases with age in many organisms, for example, in nematode [2], human [22], and mouse retinal pigment epithelium [23]. In Nothobranchius, LFD increased with age in liver, brain and gills [11, 15] and different rates of LFD correlate with differences in lifespan between *N. furzeri* strains and across Nothobranchius species [15, 16]. Linear increase of LFD was reported in the human myocardium [24, 25]. We found that LFD increased in *N. kadleci* almost linearly with age from week 11 to 41 leading to the estimation that 16 week-old *N. furzeri* GRZ shows LFD typical for 26 week-old *N. kadleci.* LFD is predictive of mortality risk at the population level in arthropods and at the individual level in *C. elegans* [2, 3]. The F_1_ individuals of the crossing between male GRZ and female *N. kadleci* showed heterosis, with lower LFD and higher survivorship than both parental strains. Heterosis, or hybrid vigor, describes the superiority in survival and performance of hybrid offspring over the average of both its genetically distinct parents [26]. So, these data would suggest that also in Nothobranchius LFD is correlated to mortality risk although a demonstration of this link would require longitudinal studies, such as those performed in *C. elegans* [2], which are technically not possible. Commonly, heterosis is attributed to dominance and overdominance as major causative genetic mechanisms [27], and also to epistatic interactions [28]. Emerging views suggest the role of non-additive gene expression, small RNAs, epigenetic regulation, including circadian-mediated metabolic pathways [29]. There is still no consensus on the relative contribution of genetic and epigenetic factors to heterosis [26].

### ACF in *N. kadleci* and the hybrids

Like LFD, ACF showed age-dependent increase between 16 and 33 weeks in *N. kadleci*, and 11 and 16 weeks in *N. furzeri*. Age-dependent ACF increase in the liver of *N. furzeri* and its correlation with interstrain differences in lifespan was described [14], and ACF in the liver also increase during aging of Medaka [30]. Unlike LFD, however, there was a drop in ACF in *N. kadleci* between 33 and 41 weeks. A possible explanation for this phenomenon could be that animals that reached this age (23% survivorship) were selected for better phenotypic condition and were less susceptible to age-related disease, as it is the case for human centenarians [31]. As the phenomenon is also detected in GRZ, a highly-inbred strain [32], it is probably not related to genetic heterogeneity in *N. kadleci*.

### LFD and ACF have different genetic bases

The differences in age trajectories of LFD and ACF demonstrate that these age-related phenotypes are markers of different biological processes. This is also supported by the observations in hybrids. In the F_1_, ACF was intermediate between the two parental strains while LFD points to heterosis. Even more clearly, ACF and LFD were not correlated in F_0_, F_1_ and F_2_ animals. Our data argue against that in Nothobranchius*,* liver lipofuscin contributes to the induction of apoptosis as suggested in other systems such as rat [8] and human [10]. Our interpretation is that apoptosis is a marker of tissue damage. Lipofuscin, on the other hand, may not be directly linked to tissue damage and rather represent an integrative marker of age.

### QTL influencing LFD

We identified two QTL for LFD on LG1 and LG11 which together explained 10% of phenotypic variation with the standard interval mapping model and no significant improvement by multi-dimensional model. QTL on LG1 showed overdominance effect, corresponding to heterosis observed in both LFD and lifespan analyses. QTL on LG11 showed additive effect. Counter-intuitively, in both loci the *N. kadleci* allele is associated with more lipofuscin than the *N. furzeri* one. Transgressive QTL segregation (phenotype effects that are extreme, novel or opposite relative to parental lines) is a described phenomenon in animals [33, 34]. Epigenetic mechanisms such as miRNAs, siRNAs, DNA methylation patterns and histone marks are suggested as causes of the heterotypic phenotypes [35, 36]. We detected no evidence of QTL interaction between the two loci. Interestingly, a suggestive QTL accounting for 6% of variation in lifespan was previously detected on LG11 [18] which corresponds to the same region of LG1 in this study. However, owing to a large confidence interval in the lifespan study, it is not possible at this stage to tell if lifespan and lipofuscin QTL actually overlap or represent different QTL on the same chromosome.

### QTL affecting ACF

With the standard interval mapping model we identified one QTL with genome-wide support on LG4 accounting for 9% of variation in ACF. Multi-dimensional scans localized three QTL on LGs 4, 6 and 10, and two suggestive QTL on LGs 7 and 15, jointly explaining 19% of the variation. These QTL comprised loci with dominance and underdominance effects in the F_2_ (Table 2, Fig. 5). The KK genotype was generally associated with lower ACF as expected. However, QTL peak location on LG4 shifted from ARCHEASE (in single QTL model) to *ERG28* (in multi-QTL model), leaving open the possibility of interacting QTL on this LG. Further, two of the QTL (LG4 and LG10) revealed epistatic interaction. In general, ACF percentage was a better trait for QTL mapping in our study probably because it is a marker of a specific biological process with a more simple genetic architecture (i.e. smaller number of loci with larger effects).

### Mapping sensitivity and resolution

Identification of QTL depends on the magnitude of their phenotypic effect. With smaller effects of QTL, a large mapping panel is required [37]. Computer simulations [38] suggested that with a population of 200 back cross or F_2_, QTL that explain at least 5% of total variance could be detected, and that on average, a QTL with 5% or 10% explained variance is mapped on an interval of 40 or 20 cM, respectively. In the present study, we have phenotypic data available for 287 individuals which are low for genetically complex phenotypes. Indeed, the identified QTL explain less than 20% of the observed LFD and APCs variances which indicates that multiple QTL with small effects remained below the power of detection in the present study [39]. This could also explain inability to detect loci where low LFD is associated with the *N. kadleci* allele. Further, a significant amount of segregation distortion was observed in this study. Segregation distortion biases the estimates of recombination fractions in genomic regions affected by segregation distortion loci (SDL), thereby not only affecting map length or marker order in those regions, but also discovery of QTL that are linked to SDL if mapping population is small [40, 41]. Segregation distortion is detrimental to the power of detecting QTL with dominance effects, while not affecting, or even aiding the identification of QTL with additive effects [42]. Furthermore, differential transmission of particular allelic combinations may lead to the evolution of reproductive isolation [43], which we suspect to be developing between *N. kadleci* and *N. furzeri*.

For ACF, multi-dimensional methods led to significant improvements in explained variance. Single-QTL methods are generally effective in detecting multiple loci underlying quantitative traits, partly because they are often located on separate chromosomes, thus independent assortment facilitate their marginal effects to be large enough to be detected [44]. However, QTL detected by this approach often represent a fraction of the total set of QTL involved in a trait. Single-QTL methods, as our ACF case suggests, in fact, ignore QTL of minor and intermediate effects, which may be linked or epistatic such that genotype at one QTL can dramatically influence QTL effects at other loci [44, 45]. In addition, interacting QTL can reduce the contributions of single significant loci [46] and explain low power in our single-QTL model.

Analysis of ACF suggested additive distribution in F_1_ and F_2_ hybrids. QTL analysis showed additive (LG6: *PHB*, LG7: *GSHR*, and LG15: *PHPT1*, Fig. S2) and underdominance (LG4: *ERG28*, LG10: *HECTD3*) effects. We speculate that additive distribution in ACF resulted from averaging on loci with effects pointing into opposite directions, particularly if more overdominant QTL were missed. Moreover, we detected evidence of opposing interaction effects between QTL on LG4 and LG10 with strong dominance-additive (d:a) and additive-dominance (a:d) effects as opposed to additive or dominance effects on both loci. Also, detection of suggestive QTL on LG7 with Bayes interval covering the entire LG suggested presence of two QTL with opposite effects (*GSHR* and *PLXNA4*, Fig. S2). These results signify a greater number of QTL of minor effects contributing to ACF variation that would require a large mapping population and dense linkage map to locate.

However, the number of QTL detected for ACF and our ability to correctly detect overdominance effects for LFD corresponding to heterosis of F_1_ population underscores the point that mapping populations of ~300 individuals may be sufficient to unravel substantial aspects of the genetic bases of complex age-related traits in *Nothobranchius* spp. This is also shown by our earlier work, where four QTL for *N. furzeri* lifespan were identified with 284 F_2_ fish [18]. Additionally, the fact that the two independently obtained genetic maps are largely consistent in LGs and marker order (Fig: S4) imply cross-species utility of maps for closely related Nothobranchius species.

## METHODS

### Fish culture and genetic crossing

Populations of *N. furzeri* and *N. kadleci* were reared in 40 L tanks in groups of, at most, 20 fish. Typically, 3-5 females were mated to 1-2 males for both species. Eggs were collected twice a week and stored at 26 °C on moist peat moss. Hatching was done when embryos showed clearly defined eyes having a gold ring around them visible in the egg. Fry were fed with brine shrimps, *Artemia* spp for 4 – 6 weeks and gradually transitioned to live *Chironomus* spp.

The mapping population was raised from the mating of one male *N. furzeri* GRZ with one female *N. kadleci* Fig. 1. Eggs for the cross were collected every second day. Thirteen F_1_ pairs were intercrossed to generate the F_2_ which were used for genetic mapping. Two progeny shared a single 5-L tank in individual compartments, from the age of about 3 weeks.

### Sample processing

Tissues for DNA and histological analysis were collected from each fish. DNA was isolated from muscle and skin tissue obtained from the area dorsal to the vent. Whole liver tissues were dissected and fixed in 4% paraformaldehyde (PFA) for protein stabilization for at least 24 hours and transferred to 50% ethanol for at least 12 hours before further processing. Automated tissues processing was conducted overnight using the Shandon Excelsior (Thermo Electron Corporation). Tissues were dehydrated in a graded ethanol series (70%, 95% and 100%); ethanol cleared in xylene; and infiltrated with paraffin. Processed tissues were embedded in paraffin using tissue lock cassettes. For all experiments, 6 µm sections were prepared with a microtome (Micron HM355S), deparaffinized with an automated system (Leica Autostainer XL, Leica) running the program: xylene 10 min, xylene 10 min, 100% EtOH 1 min, 100% EtOH 1 min, 95% EtOH 1 min, 70% EtOH 1 min, 50% EtOH 1min, wash in deionized water 2 min.

### Phenotype scoring

Lipofuscin was quantified from 40x autofluorescent images acquired at 92 ms exposure time. Per animal, 10 - 15 images were acquired in ZVI format and exported to conventional formats (PNG or JPEG) using the AxioVision software (Release 4.8). Images were batch pre-processed with a scripted action in Photoshop CS4 (ADOBE) as follows: convert to CMYK colour scheme, split channels, retain cyan channel in gray scale, threshold with a factor of 128. Autofluorescent erythrocytes were manually removed with the eraser tool.

Apoptosis measured using the terminal deoxynucleotidyl transferase (dUTP) nick end labelling (TUNEL) assay. *In situ* cell death detection kit, TMR red (Roche version April 2006) was used to stain apoptotic cells following the manufacturer's protocol for difficult tissues except that goat serum was used in the blocking step instead of bovine serum. Sections were counterstained with a DAPI fluoro-shield to facilitate normalization. At least 5 images at 20x magnification were acquired for each animal, each image being a stack of the DAPI, red, and green channels.

For both phenotypes, images were acquired only from regions not showing obvious pathology. Lipofuscin was quantified as area of granules per field (1388 × 1040 pixels) using a modified pipeline automated in CellProfiler 2.0 (BROAD Institute) for batch processing. Objects of size ≥11 pixels were included in analysis. For apoptosis, numbers of nuclei per field in DAPI channels, and numbers of cells positive for apoptosis in the corresponding red channels were counted in CellProfiler using an optimized pipeline. For each animal a phenotype value was a DAPI normalized average over all images.

### Marker generation, genotyping and variation analysis

To identify a genome-wide set of SNVs for the present study, we re-sequenced 441 gene-associated loci previously described in *N. furzeri* [32] in five *N. kadleci* and the founder pair of this cross. An additional set of SNVs was identified by intronic primers of further 108 protein-coding loci, designed using Primer3, and purchased from Eurofins MWG (Ebersberg, Germany). These loci were amplified in four each of *N. kadleci* and *N. furzeri* MZM 0403 specimens. A complete primer list is given in Table S5. Genotyping was done using the MassARRAY Sequenom technology (Sequenom, Inc.), with a list of Sequenom primers shown in Table S6.

### Linkage analysis

Genotypes were analyzed in three steps using the qtl library in R software environment [47]. First, a quality control screen was performed as follows: exclusion of markers and individuals with ≤ 50% of genotypes, checking conformity of markers and individuals to 1:2:1 genotype frequencies, inspection of observed cross-overs per individual and pairwise marker associations. Second, a linkage map was constructed. Linkage groups (LGs) were formed at a conservative initial LOD threshold 8.0 and final inclusion of weakly linked markers at minimum LOD threshold 5. Identified LGs were checked for overall pairwise linkage by plotting their estimated recombination fractions against LOD values. Genotyping error rate for the final map was estimated as log likelihood for each LG. The resulting map was compared with a previously published map [18] and finally used to scan QTL for LFD and ACF.

### QTL analysis

First, we checked correlation between each phenotype with potential covariates including gender, size (mass, length), and breeder family, and also possible correlation between the phenotypes themselves. The search for QTL was conducted using qtl and mqm libraries in R. We analyzed phenotypes separately using a two-step approach. The standard interval mapping model which assumes presence of a single QTL per LG, with additive and dominance effects, was first fitted. The loci detected with this model were thereafter used as initial factors in a multi-dimensional model in R/qtl. Additionally, we confirmed results using the automated model selection algorithm implemented in R/mqm. QTL were deemed significant if the *F*-statistic exceeded a threshold computed by permutation test with 1000 replicates. QTL confidence intervals (CI) were calculated using the Bayes intervals which are reportedly more accurate in providing the 95% CI than conventional bootstrap or 1.5-LOD intervals [48, 49].

### Statistical analysis

Phenotype summaries, testing and survival analysis were conducted in STATA 11 software (STATA Corp.). Phenotype data were tested for normality using the Shapiro-Wilk test. The *t*-test was used test for age-dependent increase for both phenotypes in parental populations. The distributions of F_1_ and F_2_ were skewed and the log transformation was identified as appropriate using the “ladder” and “gladder” commands in STATA. The Mann-Whitney test was used to test for significance in mean differences. Spearman's rank correlation was used to assess correlation between each phenotype and covariates. Kaplan-Meier survival estimates were used to describe lifespan of *N. kadleci*, and the Log-rank statistic was used to test for significant differences in survival between *N. furzeri*, *N. kadleci* and the offspring populations.

## SUPPLEMENTARY FIGURES


