# Supplementary material for: The age related markers lipofuscin and apoptosis show different genetic architecture by QTL mapping in short-lived Nothobranchius fish
Source: Aging (Albany NY). 2014 May 12;6(6):468–80. doi: 10.18632/aging.100660 (PMC4100809; doi:10.18632/aging.100660)
Supplement: Supplementary file 1 [file aging-06-468-s001.pdf]

SUPPLEMENTARY FIGURES

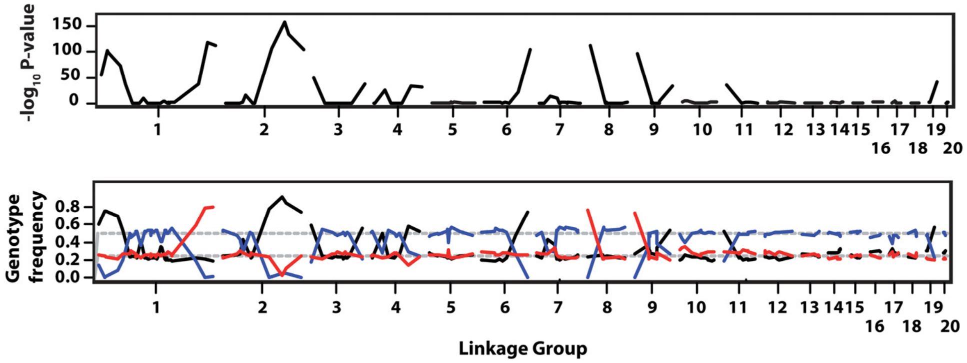

Figure S1. Transmission ratio distortion QTL. The top panel shows LGs (*x*-axis) at which distortion QTL were observed. The bottom panel shows corresponding genotype frequencies across LGs.

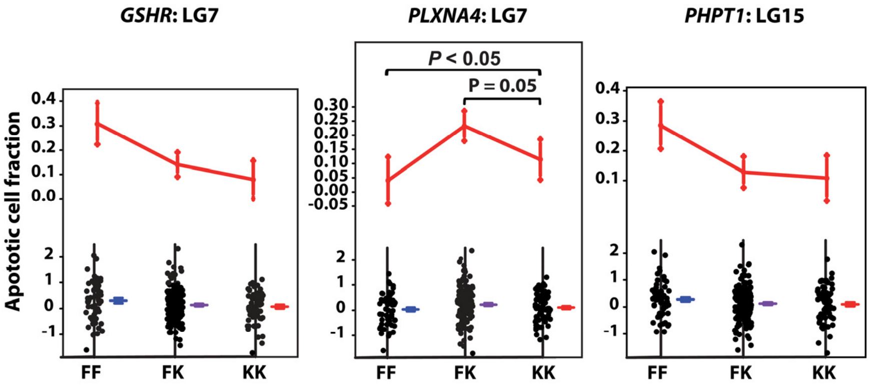

Figure S2. Allelic effects for suggestive QTL of ACF. Two QTL effects with additive and under-dominance effects were detected at LG7, and one with additive effect on LG15. The *x*-axis depicts the three genotypes at each marker – FF refers to F_2 homozygous for *N. furzeri* GRZ allele, FK stands for heterozygous F_2 and KK for F_2 homozygous for the *N. kadleci* allele. Dot plots below each effect plot shows genotype-specific data dispersion at each QTL with mean shown for each. Error bars at each genotype in are standard error of mean.

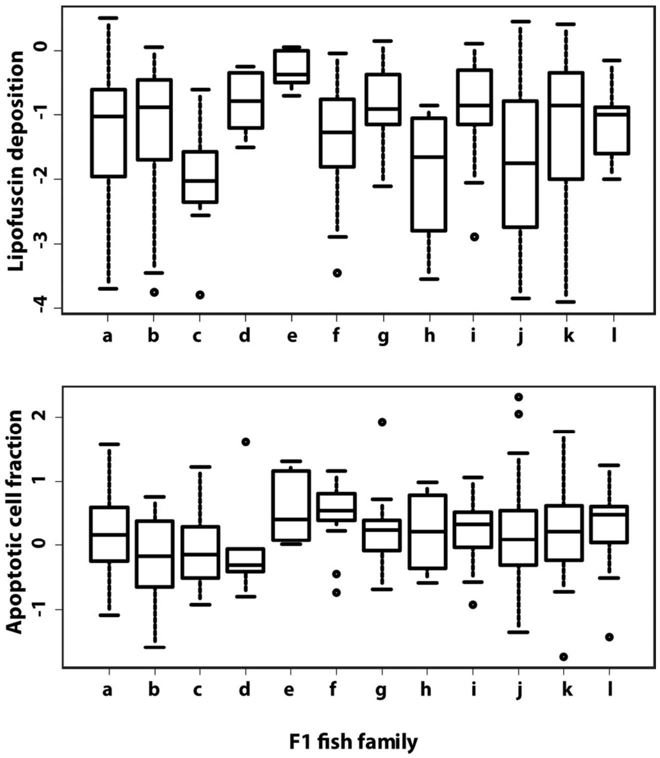

Figure S3. LFD and ACF in F_1 fish families. Both lipofuscin deposition (top) and apoptotic cell fraction (bottom) were quantified within each F_1 fish family, showing greater within-family variance in LFD compared to ACF.
